# Gender Difference in Associations between Chronic Temporomandibular Disorders and General Quality of Life in Koreans: A Cross-Sectional Study

**DOI:** 10.1371/journal.pone.0145002

**Published:** 2015-12-16

**Authors:** Tae-Yoon Kim, Joon-Shik Shin, Jinho Lee, Yoon Jae Lee, Me-riong Kim, Yong-jun Ahn, Ki Byung Park, Deok-Sang Hwang, In-Hyuk Ha

**Affiliations:** 1 Jaseng Spine and Joint Research Institute, Jaseng Medical Foundation, Seoul, Republic of Korea; 2 Department of Korean Medicine Obstetrics & Gynecology, Kyung Hee University, Seoul, Republic of Korea; University of Geneva, SWITZERLAND

## Abstract

**Background:**

Chronic temporomandibular disorder (TMD) is known to have strong correlations with psychological factors and to display gender disparity. However, while chronic TMD is known to affect quality of life, large-scale studies investigating the influence on quality of life by gender are scarce.

**Methods:**

This cross-sectional study assessed the data of 17,198 participants aged ≥19 years who completed chronic TMD and EuroQol-5 Dimension sections in the 4^th^ Korean National Health and Nutrition Examination Survey (2007–2009). We adjusted for covariates (health behavior, sociodemographic factors) in regression analysis for complex sampling design to calculate regression coefficients and 95% CIs for gender difference in the association between chronic TMD and quality of life. We also evaluated which covariates of somatic health, mental health, health behavior, and sociodemographic factors weakened the relationship between TMD and EQ-5D.

**Results:**

Prevalence of chronic TMD was 1.6% (men 1.3%, women 1.8%), and chronic TMD persisted to negatively impact quality of life even after adjusting for confounding variables. Low sociodemographic factors and health behavior had a negative effect on quality of life. Somatic health and mental health were most affected by chronic TMD. As for quality of life, women were affected to a greater extent than men by TMD. Women were more affected by osteoarthritis and general mental health (stress, depressive symptoms, and thoughts of suicide), and men by employment.

**Conclusions:**

These results imply that chronic diseases and psychological factors are important in chronic TMD, and that there may be physiological and pathological gender differences in TMD.

## Introduction

Temporomandibular disorder (TMD) is a general term that encompasses a heterogeneous group of musculoskeletal and psychophysiological pain conditions involving the temporomandibular joint (TMJ) and adjacent structures [1]. Prevalent clinical signs include pain, tenderness upon palpation, limited range of movement, and clicking sounds [2]. A wide variety of symptoms including headache, bruxism, difficulty opening the mouth, clicking sounds, and orofacial pain has been reported [3]. TMJ pain is relatively common, afflicting about 15% of women and 8% of men [4].

Pain is the most characteristic feature in most TMD cases, and the main reason patients seek treatment [5]. TMD is generally regarded as a chronic pain condition, and most cases present with considerable similarities [6]. High levels of repetitive chronic pain are known to impact quality of life [7], and of chronic disorders, chronic TMD is known to incur considerable pain, negatively influencing social and vocational function. Meanwhile, the etiology of TMD remains a highly controversial issue, as these disorders are considered to be triggered by various psychophysiological disturbers [8–11]. However, very few studies have investigated the relationship between chronic TMJ/orofacial pain and quality of life.

Murray et al. reported the health-related quality of life (HRQoL) in patients referred to a craniofacial pain clinic due to TMD and facial pain measured with the Oral Health Impact Profile [12]. Of respondents, 29.7% reported frequent sleep disturbance due to oral conditions, and 36.4% feelings of depression regarding pain-related disability and HRQoL. In a more recent systematic review on the relationship between oral health and HRQoL, only 1 study was associated with TMD [13]. A 1989 study by Reisine and Weber followed 30 TMD patients for 6 months and found that pain persisted in many patients during this period, and that there were no significant improvements in oral functionality factors even in reduction of pain [14]. Tjakkes et al. reported that while quality of life scores in TMD patients within 1 year of pain occurrence was higher than in the general population, such trends were reversed in patients with longer periods of pain [15]. These are the few studies to investigate the association between TMD and quality of life, most of which assessed oral HRQoL, as opposed to general quality of life, in small samples. To our knowledge, there are currently no national-level, large-scale studies on the relationship between TMD and quality of life. Studies investigating which factors act as mediators in the relationship between TMD and quality of life are also warranted.

The main aims of this study were to (a) use a cross-sectional design to examine whether the prevalence of chronic TMD has a negative association with quality of life; and (b) identify possible mediators in the relationship between chronic TMD and EQ-5D scores.

## Materials and Methods

### Study population and sampling

This study used data obtained from the 4^th^ Korean National Health and Nutritional Examination Survey (KNHANES) (2007–2009) conducted by the Korean Ministry of Health and Welfare. The survey consisted of three sections (the health survey, nutrition survey, and health examination) and used a rolling sampling design with a complex, stratified, multistage, probability-cluster survey in a nationally representative sample of South Koreans. More information is available in ‘‘The 4th (2007–2009) KNHANES Sample Design” and the 1st-3rd Sample Design reports. Data is made available upon email request on the KNHANES website [16]. Out of the target population of 31,705 potential surveyees, 23,632 participated in the 4^th^ KNHANES (74.5% of total target population of 31,705). After exclusion of data of 5,226 participants aged <19 years, 1,095 participants missing information on chronic TMD prevalence, and 113 participants missing EuroQol (EQ) values, data of 17,198 participants was analyzed. Missing values in data of the 17,198 respondents were excluded in corresponding adjustments for confounding variables.

### TMD

Chronic TMD was defined as self-reported experience of temporomandibular pain persisting 3 months or longer during the past year. Ever-TMD was defined as ever-experience of TMD at time of KNHANES survey.

### EQ-5D

EuroQol-5 Dimension (EQ-5D) is a HRQoL assessment tool widely used in the health and medical care sector. Outcomes range from ‘health worse than death’, represented with -1, to ‘perfect health’, represented with 1 [17]. EQ-5D consists of 5 dimensions regarding current health state, which are mobility (M), self care (SC), usual activities (UA), pain/discomfort (PD), and anxiety/depression (AD), and rates functionality in 3 grades (1, no problem; 2, some/moderate problem; and 3, extreme problem). We applied a weighted model based on Korean HRQoL estimates [18].

EQ–5D index=1−(0.050+0.096×M2+0.418×M3+0.046×SC2+0.13×SC3+0.051×UA2+0.028×UA3+0.037×PD2+0.151×PD3+0.043×AD2+0.158×AD3+0.050×N3)

If a grade 2, M2, SC2, UA2, PD2, and AD2 were assigned values of 1, and 0 if not. If a grade 3, M3, SC3, UA3, PD3, and AD3 were assigned values of 1, and 0 if not. If any of the 5 EQ-5D items was a grade 3, N3 was assigned a value of 1, and 0 if not. If all 5 EQ-5D items was grade 1, the EQ-5D index was 1.

### Covariates

Covariates included sex, age, sociodemographic factors, health behavior, somatic health, and mental health, all of which were adjusted for in stages. For assessment of associations between TMD prevalence and quality of life, sex, age, sociodemographic factors, and health behavior were adjusted. Somatic and mental health were regarded as confounding factors to be considered in TMD, and were not included in adjustments for associations with quality of life.

Participants were categorized into male/female, and age was assessed as a continuous value. Sociodemographic factors included education, income level, occupation, urban-rural gradient, and marital status. Education level was categorized as middle school graduation or lower, high school graduation or higher, and college graduation or higher. Income level was recategorized as binary values (low or mid-low; and mid-upper or high) from quartiles by equalized average monthly household income (average monthly household income divided by household member number). Employment was assessed in accordance with the Korean Standard Occupation Classification 6^th^ revision, and then divided into employed/unemployed. Health behavior covered BMI, smoking status, alcohol consumption, and moderate physical activity. BMI was categorized into <18.5, <25, and ≥25 kg/m^2^ groups in accordance with the WHO Asia-Pacific perspective. Current smokers who had smoked 5 packs of cigarettes or more over their lifetimes were considered to be ‘smokers’, and current smokers who had smoked less than 5 packs, ex- and non-smokers as ‘non-smokers’. ‘Non-drinkers,’ were defined as those who did not consume alcohol or drank to levels where daily activities were unaffected by drinking, and ‘drinkers’ as those whose daily activities were affected by alcohol consumption at least once a month. Any of the following was considered to be moderate physical activity, or adherence to regular exercise: (a) at least 3 days of 20 minute-sessions of intense, physically demanding exercise involving heavy breathing (jogging, hiking, fast biking, etc.) during the past week; (b) at least 5 days of 30-minute moderate physical exercise sessions involving slightly labored breathing (slow swimming, doubles tennis, volleyball, etc.) during the past week; or (c) at least 5 days of 30 minute walks during the past week.

Somatic health was assessed with presence of any chronic hypertension, diabetes, asthma, and osteoarthritis of 3 months or more over the past year. Mental health was determined by presence of stress, depressive symptoms, thoughts of suicide, and sleep duration. Stress was categorized into high-stress, and low- or almost no-stress groups, and depressive symptoms were defined as 2 or more consecutive weeks of depressive symptoms. Thoughts of suicide were defined as thoughts of suicide over the past year, and sleep duration was assessed as a continuous variable.

### Statistical Analysis

KNHANES is a national-level sample survey that applies stratified cluster sampling and weighted values. We thus analyzed data using stratified, cluster, and weighted variables for complex sampling design factors in complex sampling design analysis. All analyses were performed using statistical package SAS Ver. 9.3 (SAS Institute Inc, Cary, NC, USA), and P < 0.05 was considered to be statistically significant. Continuous variables are given as mean and standard error, and categorical variables as frequency and percentage (%). Characteristics differences by chronic TMD were assessed by Rao-Scott chi-square test or t-test. Complex sampling design regression analysis adjusted for covariates was used to calculate regression coefficient and 95% confidence intervals (CIs) and assess the association between chronic TMD and quality of life. Age, sex and other covariates were included in models as separate covariates, covariate blocks, and all covariates in the full model to calculate the regression coefficient for chronic TMD.

### Ethics Statement

The surveyor was not provided any information on a participant prior to the survey, and all participants provided written consent to participate in the health survey and examination. The study was approved by the Institutional Review Boards (IRBs) of Jaseng Hospital of Korean Medicine in Seoul, Korea (IRB approval number: KNJSIRB2015-54).

## Results

Prevalence of ever-TMD was 3.2% (men 2.5%, women 3.7%), and that of chronic TMD was 1.6% (n = 273, men 1.3%, women 1.8%) of the 17,198 respondents.

Univariate analysis showed that age, sex, education level, marital status, chronic hypertension, chronic diabetes, and stress level were associated with chronic TMD prevalence (Table 1).

**Table 1:** Characteristics of participants aged ≥19 years of KNHANES Ⅳ, 2007 to 2009 (N = 17,198).

		Chronic TMD		
Factors		No (n = 16,925)	Yes (n = 273)	p-value^a^
Age (mean±SE) (years)		44.7±0.25	33.9±0.81	< .0001
Sex				
	Male	7196 (98.7)	95 (1.3)	0.1753
	Female	9729 (98.2)	178 (1.8)	
Household income level				
	Low or mid-low	8239 (98.5)	125 (1.5)	0.3353
	Mid-upper or high	8265 (98.3)	141 (1.7)	
Education level				
	Middle school graduation or lower	6870 (99.3)	46 (0.7)	< .0001
	High school graduation or higher	5783 (97.9)	123 (2.1)	
	College graduation or higher	4253 (97.6)	104 (2.4)	
Employment state				
	Unemployed	5706 (98.2)	102 (1.8)	0.1467
	Employed	9722 (98.4)	156 (1.6)	
Marital status^b^				
	Unmarried	2219 (96)	92 (4.0)	< .0001
	Married	14641 (98.8)	181 (1.2)	
Urban-rural gradient^c^				
	Eup/Myeon	4505 (98.9)	50 (1.1)	0.0598
	Dong	12420 (98.2)	223 (1.8)	
Smoking status				
	Non-smoker	13211 (98.4)	217 (1.6)	0.9638
	Smoker	3687 (98.5)	56 (1.5)	
Influence of alcohol consumption on daily activities				
	No	14642 (98.5)	221 (1.5)	0.0546
	Yes	2267 (97.8)	52 (2.2)	
Body mass index (kg/m2)				
	<18.5	775 (97.5)	20 (2.5)	0.1042
	<25	10694 (98.3)	183 (1.7)	
	≥25	5364 (98.8)	68 (1.3)	
Adherence to regular exercise				
	No	7373 (98.4)	118 (1.6)	0.5736
	Yes	9428 (98.4)	152 (1.6)	
Hypertension				
	No	13765 (98.2)	255 (1.8)	< .0001
	Yes	3160 (99.4)	18 (0.6)	
Diabetes				
	No	15757 (98.4)	264 (1.7)	0.0442
	Yes	1168 (99.2)	9 (0.8)	
Asthma				
	No	16561 (98.4)	262 (1.6)	0.1999
	Yes	364 (97.1)	11 (2.9)	
Osteoarthritis				
	No	14809 (98.4)	234 (1.6)	0.3495
	Yes	2116 (98.2)	39 (1.8)	
Stress level				
	Low or almost no stress	12129 (98.7)	163 (1.3)	0.0002
	High stress	4781 (97.8)	110 (2.3)	
Depressive symptoms				
	No	14259 (98.5)	211 (1.5)	0.0415
	Yes	2653 (97.7)	62 (2.3)	
Thoughts of suicide				
	No	13786 (98.5)	211 (1.5)	0.4376
	Yes	3113 (98.1)	62 (2.0)	
Sleep duration (mean±SE) (hours)		6.9±0.01	7.0±0.09	0.0687

^a^ P-value from t-test or Rao-scott chi-square test for continuous or categorical variables.

^b^ Unmarried status refers to single individuals who are not divorced or bereaved.

^c^ Eup/Myeon/Dong are administrative district divisions. Eup/Myeon are subdivisions of rural districts, and an Eup can apply for Myeon status if the area has acquired town features and a population of ≥20,000 and ≤50,000. Dong is a subdivision of urban districts.

Factors that were negatively associated with EQ-5D were higher chronicity, old age, female gender, low household income, low education level, unemployment, unmarried status, rural area residence, smoking, alcohol consumption, obesity, and not exercising regularly.

In assessing gender differences in association with quality of life, men appeared to be more affected by TMD than women. Also, while smoking status, alcohol consumption, and obesity were not significantly associated with quality of life in men, they were in women (Table 2).

**Table 2:** Association between chronic TMD and quality of life as assessed by EQ-5D in participants aged ≥19 years of KNHANES Ⅳ, 2007 to 2009.

		Total			Men			Women		
Factors		β^a^	95% CI	p-value	β^a^	95% CI	p-value	β^a^	95% CI	p-value
TMD										
	No									
	Yes	-0.025	(-0.037, -0.013)	< .0001	-0.021	(-0.039, -0.003)	0.0226	-0.027	(-0.042, -0.012)	0.0006
Age (years)		-0.003	(-0.003, -0.002)	< .0001	-0.002	(-0.002, -0.001)	< .0001	-0.003	(-0.004, -0.003)	< .0001
Sex										
	Male	0.026	(0.022, 0.031)	< .0001						
	Female									
Household income level										
	Low or mid-low	-0.012	(-0.015, -0.008)	< .0001	-0.009	(-0.014, -0.004)	0.0001	-0.014	(-0.019, -0.009)	< .0001
	Mid-upper or high									
Education level										
	Middle school graduation or lower	-0.028	(-0.034, -0.021)	< .0001	-0.027	(-0.034, -0.019)	< .0001	-0.021	(-0.03, -0.012)	< .0001
	High school graduation or higher	-0.001	(-0.005, 0.002)	0.3881	-0.004	(-0.008, 0.001)	0.0988	0.003	(-0.002, 0.008)	0.2803
	College graduation or higher									
Employment state										
	Unemployed	-0.024	(-0.029, -0.020)	< .0001	-0.038	(-0.045, -0.030)	< .0001	-0.018	(-0.024, -0.013)	< .0001
	Employed									
Marital status^b^										
	Unmarried	-0.034	(-0.039, -0.028)	< .0001	-0.012	(-0.020, -0.005)	0.0008	-0.042	(-0.050, -0.034)	< .0001
	Married									
Urban-rural gradient^c^										
	Eup/Myeon	-0.012	(-0.018, -0.006)	< .0001	-0.009	(-0.016, -0.003)	0.0035	-0.016	(-0.024, -0.007)	0.0004
	Dong									
Smoking status										
	Non-smoker	0.007	(0.002, 0.011)	0.0022	0.001	(-0.003, 0.006)	0.6283	0.021	(0.010, 0.033)	0.0003
	Smoker									
Influence of alcohol consumption on daily activities										
	No	0.007	(0.003, 0.012)	0.0006	0.004	(-0.000, 0.009)	0.0571	0.011	(0.002, 0.021)	0.02
	Yes									
Body mass index (kg/m2)										
	<18.5	0.007	(-0.001, 0.014)	0.0848	-0.009	(-0.022, 0.005)	0.1984	0.014	(0.004, 0.024)	0.0082
	<25	0.008	(0.004, 0.012)	< .0001	0.001	(-0.003, 0.006)	0.6447	0.013	(0.007, 0.019)	< .0001
	≥25									
Adherence to regular exercise										
	No	-0.009	(-0.013, -0.005)	< .0001	-0.007	(-0.012, -0.002)	0.0035	-0.011	(-0.016, -0.006)	< .0001
	Yes									

Multiple regression analysis

^a^ Regression coefficients

^b^ Unmarried status refers to single individuals who are not divorced or bereaved.

^c^ Eup/Myeon/Dong are administrative district divisions. Eup/Myeon are subdivisions of rural districts, and an Eup can apply for Myeon status if the area has acquired town features and a population of ≥20,000 and ≤50,000. Dong is a subdivision of urban districts.

After adjusting for age and sex, influence of the covariate blocks (somatic health, mental health, health behavior, and sociodemographic factors) on the association between TMD and EQ-5D was assessed (Table 3).

**Table 3:** Influence of covariates in linear regression analyses of the association between TMD and quality of life in participants aged ≥19 years of KNHANES Ⅳ, 2007 to 2009.

			Total			Men				Women		
Factors			β^b^	95% CI	p-value	β^b^	95% CI	p-value		β^b^	95% CI	p-value
Unadjusted			-0.001	(-0.015, 0.013)	0.9036	0.002	(-0.016, 0.020)	0.8460		0.000	(-0.018, 0.019)	0.9665
Age and Sex			-0.031	(-0.044, -0.018)	< .0001	-0.021	(-0.039, -0.004)	0.0182		-0.038	(-0.056, -0.020)	< .0001
	Somatic health ^a^											
		Hypertension ^a^	-0.031	(-0.044, -0.018)	< .0001	-0.022	(-0.039, -0.004)	0.0176		-0.038	(-0.056, -0.020)	< .0001
		Diabetes ^a^	-0.031	(-0.043, -0.018)	< .0001	-0.022	(-0.039, -0.004)	0.0183		-0.037	(-0.055, -0.019)	< .0001
		Asthma ^a^	-0.029	(-0.042, -0.016)	< .0001	-0.021	(-0.038, -0.003)	0.0236		-0.036	(-0.054, -0.018)	0.0001
		Osteoarthritis ^a^	-0.024	(-0.037, -0.011)	0.0003	-0.020	(-0.037, -0.002)	0.0324		-0.028	(-0.045, -0.011)	0.0016
		All somatic health ^a^	-0.022	(-0.035, -0.010)	0.0005	-0.019	(-0.037, -0.001)	0.0390		-0.026	(-0.043, -0.009)	0.0028
	Mental health ^a^											
		Stress ^a^	-0.026	(-0.039, -0.014)	< .0001	-0.019	(-0.037, -0.002)	0.0309		-0.032	(-0.049, -0.014)	0.0004
		Depressive symptoms ^a^	-0.027	(-0.039, -0.014)	< .0001	-0.020	(-0.038, -0.003)	0.0247		-0.032	(-0.049, -0.015)	0.0003
		Thoughts of suicide ^a^	-0.028	(-0.040, -0.015)	< .0001	-0.022	(-0.039, -0.004)	0.0135		-0.032	(-0.050, -0.015)	0.0003
		Sleep duration ^a^	-0.030	(-0.044, -0.017)	< .0001	-0.021	(-0.039, -0.004)	0.0188		-0.038	(-0.056, -0.019)	< .0001
		All mental health ^a^	-0.024	(-0.036, -0.012)	0.0002	-0.020	(-0.037, -0.003)	0.0219		-0.027	(-0.044, -0.010)	0.0022
	Health behavior ^a^											
		Smoking ^a^	-0.031	(-0.044, -0.018)	< .0001	-0.022	(-0.040, -0.004)	0.0166		-0.037	(-0.054, -0.019)	< .0001
		Drinking ^a^	-0.031	(-0.044, -0.018)	< .0001	-0.021	(-0.039, -0.004)	0.0184		-0.038	(-0.056, -0.020)	< .0001
		BMI ^a^	-0.030	(-0.042, -0.017)	< .0001	-0.021	(-0.039, -0.004)	0.0190		-0.036	(-0.052, -0.019)	< .0001
		Regular exercise ^a^	-0.032	(-0.044, -0.019)	< .0001	-0.022	(-0.039, -0.004)	0.0164		-0.039	(-0.057, -0.021)	< .0001
		All health behavior ^a^	-0.030	(-0.042, -0.018)	< .0001	-0.022	(-0.04, -0.004)	0.0162		-0.034	(-0.050, -0.019)	< .0001
	Sociodemographic factors ^a^											
		Household income ^a^	-0.031	(-0.045, -0.018)	< .0001	-0.024	(-0.042, -0.006)	0.0097		-0.037	(-0.056, -0.019)	< .0001
		Education ^a^	-0.031	(-0.044, -0.019)	< .0001	-0.023	(-0.041, -0.005)	0.0122		-0.038	(-0.056, -0.020)	< .0001
		Employment ^a^	-0.028	(-0.041, -0.015)	< .0001	-0.019	(-0.037, -0.001)	0.0368		-0.034	(-0.053, -0.016)	0.0002
		Marital status ^a^	-0.027	(-0.040, -0.015)	< .0001	-0.019	(-0.037, 0.000)	0.0455		-0.035	(-0.053, -0.017)	0.0001
		Uban-rural gradient ^a^	-0.031	(-0.044, -0.018)	< .0001	-0.022	(-0.039, -0.004)	0.0181		-0.038	(-0.056, -0.020)	< .0001
		All sociodemographic factors ^a^	-0.026	(-0.039, -0.013)	< .0001	-0.020	(-0.038, -0.002)	0.0296		-0.030	(-0.048, -0.013)	0.0009
All Covariates ^a^			-0.014	(-0.025, -0.003)	0.0140	-0.017	(-0.034, 0)	0.0488		-0.010	(-0.024, 0.004)	0.1556

^a^ The influence of covariates should be interpreted as the difference in the regression coefficients of chronic TMD between the model adjusted for age and sex only, and the model including the listed covariates.

^b^ All regression coefficients (β) are for the association between chronic TMD and EQ-5D, adjusted for the listed covariate.

First, regarding EQ-5D scores, the nonstandardized regression coefficient was -0.031, which decreased after adjusting for age and sex. This implies that chronic TMD negatively impacts EQ-5D scores. The nonstandardized regression coefficient was used as standard for comparison with other variables. Therefore, after including adjustments for somatic health, mental health, health behavior or sociodemographic factors, the absolute value of the difference between the two coefficients will reflect the difference in the effect sizes of each factor.

The absolute difference between -0.031 and the regression coefficient of each factor reflects its respective influence size, and the most influential factor was somatic health which decreased the association with TMD by 27%. Other influential factors included mental health, sociodemographic factors, and health behavior, which weakened the relationship with TMD by 23%, 12%, and 5%, respectively. Of the variable blocks, somatic health was most influential with a 23% effect mainly due to inclusion of osteoarthritis. Other chronic diseases were not as influential. Of individual factors, stress and depressive symptoms (included in mental health factors) were the second most prominent factors with a 14% association. In investigations on gender differences, TMD influenced women more than it did men, which had similar distributions with the whole data set. Of variable blocks, somatic health weakened associations by 31%, and mental health by 29% in women. Of individual variables, osteoarthritis decreased associations by 26%, stress by 17%, depressive symptoms by 16%, and thoughts of suicide by 15% in women. Sociodemographic factors weakened relations by 15% in men, followed by somatic health (14%), mental health (10%), and health behavior (1%). Of individual variables, occupation, marital status, stress, and osteoarthritis impacted relationships by 21%, 16%, 12% and 11%, respectively.

Upon full adjustment, association between TMD and quality of life decreased by 27% in men, and by 67% in women, showing that quality of life in men was impacted more heavily by TMD.

## Discussion

These results show that chronic TMD negatively influenced quality of life even after adjusting for sex, age, sociodemographic factors, and health behavior. Low sociodemographic factors and health behavior also generally had a negative impact on quality of life. In the association between chronic TMD and quality of life, somatic health and mental health were most influential. The prevalence of chronic and ever-TMD was higher in women, and women were more heavily influenced by chronic TMD. In relationship with quality of life, women displayed stronger associations with osteoarthritis and general mental health (stress, depressive symptoms, and thoughts of suicide), while men were influenced most by employment status.

The fact that osteoarthritis, a somatic health variable, and not mental health, was most powerful in weakening associations with TMD, was highly surprising. This may be because TMD and osteoarthritis are musculoskeletal pain disorders that share similar pathophysiological pathways, and individuals with TMD are susceptible to other musculoskeletal disorders, consequently affecting quality of life. Of the 5 EQ-5D dimensions, mobility, self care, usual activities, and pain/discomfort are likely to be easily influenced by musculoskeletal pain disorders.

Plesh et al. studied the association between TMD pain, comorbid pains and muscle disorder-type pains in a national US sample, and discovered that TMD pain does not occur separately, usually accompanied by 2 or more comorbid pains, and that this tendency is especially strong in women [19].

These results are in line with previous reports on TMD pain concurring with other common pains such as migraine/headache [20–25], neck [26–28], low back [29,30], and joint pain [31]. TMD pain showed the highest association with neck pain and migraine/headache pain, followed by back pain and joint pain. Furthermore, women reported greater number of comorbid pains, which is consistent with prior research results reporting that women show higher prevalence of certain pains compared to men [32,33].

Existing literature suggest that psychological factors are important and related with TMD, and a relationship between the onset, symptoms, prognosis and treatment of TMD and psychosocial disorders has previously been demonstrated [34–37]. Stress [38–40], anxiety [34,41,42] and depression [37,43,44] are some important psychological factors related with TMD. Giannakopoulos et al. reported that female participants with TMD pain displayed significantly more depressive symptoms compared to the general population, and males suffered more severe depressive symptoms than females under similar TMD conditions, suggesting that chronic TMD pain afflicts patients with depression regardless of gender [45]. Sherman et al. discovered that patients displayed greater improvement with concurrent psychological treatment than conventional dental care alone, implying that psychological factors are indeed a significant factor to be considered in TMD treatment [46]. Similarly, Aggarwal et al. found that a comprehensive treatment plan encompassing cognitive behavior therapy, posture regulation, and biofeedback in subjects with orofacial pain was more effective than usual care in long-term pain alleviation and stated the importance of and need for psychosocial intervention in TMD care [47].

Women appear to be more strongly influenced by mental health and psychological factors in the association with TMD. Rollman et al. broke down the gender disparity in TMD as follows: First, women sought treatment 7 times more than men for TMD [48], which reflects gender role or psychological difference between genders in seeking solutions to pain [49]. Second, men show higher sensitivity to pain than women when healthy [50]. Third, women tend to be more cautious in recognizing and accepting physical symptoms, and monitoring change than men [51]. Fourth, gender differences may be influenced by the endocrine system. According to a 1997 study by LeResche et al., TMD occurred in about 30% of menopausal women on estrogen replacement medication, hinting at an association between sex hormones and TMD occurrence [52]. Dao et al. also suggested that reproductive hormones may contribute to TMD pathophysiology based on the observation that myofascial pain is more prevalent in women with childbirth experience [53].

There are also numerous studies pointing to a relationship between lifestyle and health-related behavior and TMD, such as a study on nonspecific physical symptoms (excluding para-functional habit and pain requiring intervention) in the younger population by Rodrigues et al. [54]. In addition, Weingarten et al. concluded that current smoking had greater influence on TMD pain in 606 patients receiving treatment in the relationship between smoking and TMD symptoms [55].

Some major strengths of this study are that the health examination and surveys were systematically conducted by trained surveyors in a national-level sample of South Koreans. This is the first study to our knowledge to comprehensively assess the impact of TMD on general quality of life as opposed to oral HRQoL in a national-level study. Factors that may act as confounding variables in the association between chronic TMD and quality of life were adjusted to investigate the extent to which these factors impact TMD and quality of life.

A limitation of this study is that only associations, not causal relationships, can be drawn from the results owing to its cross-sectional design, where investigations are restricted to a certain point within a short window of time. Another limitation is that data on TMD patients was based on self-assessment and self-report in questionnaire form as opposed to physician diagnosis or diagnostic examination. Only 273 participants were found to have chronic TMD in the current study, which is 1.6% of the participant Korean population. This number is lower than the corresponding U.S. temporomandibular joint and muscle disorder prevalence of 4.6%. While TMD studies based in the U.S. employ questions widely used in other studies and examined for validity to define TMD such as “facial ache or pain in the jaw muscles or the joint in front of the ear” [56,57], KNHANES IV examined whether participants had suffered from TMJ disorders for 3 months or longer over the past year, and this difference in prevalence may reflect disparity in chronicity, survey content and method. Surveys are also susceptible to memory bias, which is an additional limitation of this study.

Several studies have been conducted to determine the principle and mechanism through which TMD physically and psychologically affects patients. Christidis et al. discovered that 5-HT3A(serotonin)-receptor, an important pain transmitter, exists in high levels in the masseter and tibialis muscles, and women with TMD possess more nerve fibers releasing 5-HT3A-receptors [58]. Also, Ojima et al. revealed that genetic factors involved in serotonin transport modulate serotonin secretion, with consequent potential to act as a catalyst in pathological expression of TMD [59]. This study holds significance in that assessment of gender-specific patterns in associations between TMD and quality of life and other covariates will help enhance understanding and management of TMD. Additional studies are warranted for further establishment of etiology and causal relationships.

In conclusion, while TMD itself is negatively associated with general quality of life, comorbidities such as osteoarthritis and mental health issues strengthen this negative relationship. The association was stronger in women, which may reflect susceptibility to chronic pain and mental health disorders. Further items that may be of interest include association of quality of life with severity or prevalence period of TMD symptoms, and gender analysis of the various pathological states that may accompany TMD.
